# The efficacy and safety of β-nicotinamide mononucleotide (NMN) supplementation in healthy middle-aged adults: a randomized, multicenter, double-blind, placebo-controlled, parallel-group, dose-dependent clinical trial

**DOI:** 10.1007/s11357-022-00705-1

**Published:** 2022-12-08

**Authors:** Lin Yi, Andrea B. Maier, Rongsheng Tao, Zhigang Lin, Aditi Vaidya, Sohal Pendse, Sornaraja Thasma, Niranjan Andhalkar, Ganesh Avhad, Vidyadhar Kumbhar

**Affiliations:** 1 Abinopharm, Inc, 3 Enterprise Drive, Suite 407, Shelton, CT 06484 USA; 2 Department of Human Movement Sciences, @AgeAmsterdam, Amsterdam Movement Sciences, Vrije Universiteit Amsterdam, Van der Boechorststraat 7, 1081 BT Amsterdam, The Netherlands; 3 Healthy Longevity Translational Research Program, Yong Loo Lin School of Medicine, National University of Singapore, Singapore, 119228 Singapore; 4 Centre for Healthy Longevity, @AgeSingapore, National University Health System, 28 Medical Drive, Singapore, 117456 Singapore; 5 Huzhou Yihui Biotechnology Co., Ltd, 1366 Hong Feng Road, Huzhou, Zhejiang 313000 People’s Republic of China; 6 ABA Chemicals Corporation, 67 Libing Road, Building 4, Zhangjian Hi-Tech Park, Shanghai, 201203 People’s Republic of China; 7 ProRelix Services LLP, 102 A/B, Park Plaza, Karve Road, Karve Nagar, Pune, Maharashtra 411052 India; 8 Lotus Healthcare & Aesthetics Clinic, 5 Bramha Chambers, 2010 Sadashivpeth, Tilak Road, Pune, Maharashtra India; 9 Sunad Ayurved, Siddhivinayak Apart, Jeevan Nagar, Maharashtra 411033 Chinchwad, Pune, India

**Keywords:** Nicotinamide mononucleotide, NMN, NAD, Randomized clinical trial, Middle aged, Healthy, Physical functional performance, Longevity, Safety, Geroscience

## Abstract

In animal studies, β-nicotinamide mononucleotide (NMN) supplementation increases nicotinamide adenine dinucleotide (NAD) concentrations and improves healthspan and lifespan with great safety. However, it is unclear if these effects can be transferred to humans. This randomized, multicenter, double-blind, placebo-controlled, parallel-group, dose-dependent clinical trial included 80 middle-aged healthy adults being randomized for a 60-day clinical trial with once daily oral dosing of placebo, 300 mg, 600 mg, or 900 mg NMN. The primary objective was to evaluate blood NAD concentration with dose-dependent regimens. The secondary objectives were to assess the safety and tolerability of NMN supplementation, next to the evaluation of clinical efficacy by measuring physical performance (six-minute walking test), blood biological age (Aging.Ai 3.0 calculator), Homeostatic Model Assessment for Insulin Resistance (HOMA-IR), and subjective general health assessment [36-Item Short Form Survey Instrument (SF-36)]. Statistical analysis was performed using the Per Protocol analysis with significant level set at *p* = 0.05. All 80 participants completed the trial without trial protocol violation. Blood NAD concentrations were statistically significantly increased among all NMN-treated groups at day 30 and day 60 when compared to both placebo and baseline (all *p* ≤ 0.001). Blood NAD concentrations were highest in the groups taking 600 mg and 900 mg NMN. No safety issues, based on monitoring adverse events (AEs), laboratory and clinical measures, were found, and NMN supplementation was well tolerated. Walking distance increase during the six-minute walking test was statistically significantly higher in the 300 mg, 600 mg, and 900 mg groups compared to placebo at both days 30 and 60 (all *p* < 0.01), with longest walking distances measured in the 600 mg and 900 mg groups. The blood biological age increased significantly in the placebo group and stayed unchanged in all NMN-treated groups at day 60, which resulted in a significant difference between the treated groups and placebo (all *p* < 0.05). The HOMA-IR showed no statistically significant differences for all NMN-treated groups as compared to placebo at day 60. The change of SF-36 scores at day 30 and day 60 indicated statistically significantly better health of all three treated groups when compared to the placebo group (*p* < 0.05), except for the SF-36 score change in the 300 mg group at day 30. NMN supplementation increases blood NAD concentrations and is safe and well tolerated with oral dosing up to 900 mg NMN daily. Clinical efficacy expressed by blood NAD concentration and physical performance reaches highest at a dose of 600 mg daily oral intake. This trial was registered with ClinicalTrials.gov, NCT04823260, and Clinical Trial Registry - India, CTRI/2021/03/032421.

**Supplementary Information:**

The online version contains supplementary material available at 10.1007/s11357-022-00705-1.

## Introduction

β-Nicotinamide mononucleotide (NMN) is a natural product which exists in small quantity in most plants, such as edamame, broccoli, and cucumber [1]. It is also an endogenous molecule in all mammalian tissues [2, 3]. Its biological functions are linked to the ability to boost nicotinamide adenine dinucleotide (NAD), a product of the salvage pathway in NAD biosynthesis in which NMN serves as a precursor [2, 3]. NAD concentration declines with age affecting mammalian longevity and age-related health conditions through its functions of energy metabolization and activations of poly ADP-ribose polymerase (PARP), Sirtuin proteins, and etc. in mammalian tissues [2, 4].

Many preclinical studies on NMN supplementation have been reported [2–4]. NMN treatment of Werner syndrome (WS) worms and flies with 1mM dosing elevated NAD concentrations 2.2 times higher than control and extended lifespan to 19.8 days vs. 13.9 days in control [5]. NMN intervention to healthy wild-type 3-4-month C57BL/6N mice steadily increased liver NAD concentrations over 30 min after a single oral 300 mg/kg dose, and effectively mitigated age-associated physical decline and was well tolerated after a 12-month oral treatment of 100 mg/kg/day or 300 mg/kg/day [1]. Clinical benefits of NMN supplementation in mouse models have shown doubled running endurance on aging mice [6], restored cognition in Alzheimer’s disease rat model [7], and reversed vascular dysfunction in old mice [8]. However, human clinical trials with NMN are limited [9–17]. The first human clinical trial confirmed that NMN supplementation was safe up to 500 mg/day and plasma concentrations of NAD metabolites were significantly increased, but the changes of plasma NAD concentration were not mentioned, and the trial was an open-label single oral dosing with only 10 healthy male participants [9]. The first randomized controlled trial (RCT) of NMN supplementation revealed the increases of skeletal muscle insulin sensitivity and signaling, and significant elevation of NAD concentration in peripheral blood mononuclear cell (PBMC), but found no significant change in muscle NAD concentration, and the trial used only a fixed 250 mg/day oral dose with prediabetic and obese female participants [10]. Another RCT found that the NMN supplementation was safe, but the increase of blood NAD concentration was not statistically significant with 300 mg/day oral dose on 62 middle-aged healthy men and women for 60 days [11]. A similar RCT reported that oral 300 mg/day NMN supplement was safe and significantly improved several blood biomarkers, such as HbA1c and HDL-C, but has significantly reduced blood NAD level for the 17 postmenopausal female participants after an 8-week trial [12]. A 12-week RCT with a fixed 250 mg/day oral NMN dose on 42 healthy older men showed that blood NAD concentration was significantly elevated and NMN was well tolerated, but NMN supplementation produced mixed results on skeletal muscle strength tests [13]. A separate RCT disclosed that, after intervention also with a fixed 250 mg/day NMN oral dose for 12 weeks on 30 healthy males and females, blood NAD concentration increased significantly starting at week 4 until the end of treatment at week 12 and returned to original level 4 weeks after NMN treatment, and the NMN supplementation was safe and well tolerated [14]. An RCT with 1000 and 2000 mg/day oral doses of MIB-626, a unique microcrystalline NMN formulation, on 32 overweight or obese older adults showed that blood NAD concentration was dose-dependently increased and NMN treatment was also safe and well tolerated, but the trial found that lower doses failed to significantly elevate blood NAD concentrations [15]. A 6-week RCT with healthy amateur athletes with 300, 600, and 1200 mg/day NMN oral regimens concluded that exercise plus NMN supplementation was significantly and dose-dependently increased aerobic capacity but found no impact on physical strength when compared to exercise alone [16]. Another RCT on the effects of the time-dependent intake (morning or afternoon) of NMN on sleep quality, fatigue, and physical performance in older adults was conducted with 250 mg/day NMN oral treatment for 12 weeks [17]. The trial found that only drowsiness and lower limb function in the afternoon NMN-treated group were significantly improved among the many trial end points measured. In summary, NMN supplementation was found safe and well tolerated in all the above disclosed human clinical trials [9–17]. However, only mixed results were reported on the effects of NMN to human NAD elevation [9–15] and age-related physical decline and health conditions [9–13, 16, 17]. Most of these studies used either 250 or 300 mg/day fixed dosage [10–14, 17] or only recruited either male [9, 13] or female participants [10, 12] or participants with certain health conditions such as obese and overweight [10, 15]. Further clinical studies are needed to evaluate the effects of NMN supplementation on healthy middle-aged adults of both males and females with dose-dependent oral dosing regimens in the 300-900 mg/day range on the effects of NAD concentration, safety and tolerability, and age-related physical decline and health conditions.

## Materials and methods

### Study design, ethical approval, and participants

This multicenter, randomized, parallel, double-blinded, placebo-controlled, dose-dependent clinical trial on NMN supplementation at daily oral doses of 300 mg, 600 mg, and 900 mg for 60 days aimed to test the effect of NMN supplementation on blood NAD concentration (primary outcome), safety, tolerability, and clinical efficacy (secondary outcomes) of NMN supplementation.

This clinical trial was conducted in accordance with the ethical principles laid down by Declaration of Helsinki (Taipei 2016), the principles of ICH Guidelines for Good Clinical Practice (GCP) (1997), and New Drugs and Clinical Trial Rules, 2019. The “Royal Ethics Committee, Pune, India” reviewed and approved the clinical trial protocol and informed consent form submitted by the two principal investigators (PI) of this trial. The conduct of trial-related activities commenced after the approval from the ethics committee. The “Royal Ethics Committee, Pune, India” is an independent ethics committee formed as per the “New Drugs and the Clinical Trials Rule 2019”. The ethics committee is duly registered with Drugs Controller General of India (DCGI) via number - ECIV45/Indt/MII/2013/RR-19. This trial was registered with ClinicalTrials.gov, NCT04823260 and Clinical Trial Registry - India, CTRI/2021/03/032421. The trial was monitored by ProRelix Services LLP, a clinical research organization (CRO), Pune, India.

The trial was conducted at two clinic centers: Lotus Healthcare and Aesthetics Clinic, and Sunad Ayurved (both in Pune, India), with an experienced principal investigator (PI) at each site. Recruitment was facilitated by targeted advertisement to healthy volunteers from the PIs’ own databases and referrals of other physicians at the two centers. Each volunteer was firstly given trial information and signed an informed consent form (ICF) before screening. The screening process entailed demographic data (name, sex, date of birth, age, race, height, weight) and medical history. Physical examinations including general and systemic examinations and vital signs (blood pressure, pulse rate, respiration rate, systolic and diastolic pressure, and body temperature), electrocardiogram (ECG), and X-ray of chest were performed on each volunteer. All volunteers were symptomatically assessed for coronavirus disease 2019 (COVID-19). Blood and urinary samples were taken at the two clinical centers, stored, and transported to Suburban Diagnostics (Pune, India) by following standard operation procedure (SOP) according to good clinical practice (GCP). Suburban Diagnostics (Pune, India) conducted the clinical lab tests which included hematology [hemoglobin, hematocrit, white blood count (WBC) (total and differential), red blood cell count (RBC), platelet count, prothrombin time (PT), activated partial thromboplastin time (aPTT), mean corpuscular hemoglobin concentration (MCHC), and mean corpuscular volume (MCV)], clinical chemistry [blood glucose (random), serum triglyceride, low-density lipoprotein (LDL), high-density lipoprotein (HDL), total cholesterol, serum creatinine, urea, aspartate aminotransferase (AST), alanine aminotransferase (ALT), alkaline phosphatase, total bilirubin, sodium, blood urea nitrogen (BUN), glomerular filtration rate (GFR), uric acid, chloride, calcium, potassium, albumin, total proteins], and urinalysis (pH, specific gravity, protein, glucose, ketone bodies, leukocytes, nitrite, hemoglobin/erythrocytes, urobilinogen, and bilirubin). For female volunteers, urine pregnancy test was assessed.

Inclusion criteria for participants were 40-65 years and healthy volunteers of both males and females, body mass index (BMI) between 18.5 and 35 kg/m^2^, not taking any supplements containing any form of niacin for 7 days prior to baseline and for the duration of the trial, being able to maintain consistent diet and lifestyle habits throughout the trial, willing to agree to use effective contraceptive methods throughout the trial, willing to provide written informed consent, willing to follow verbal and written trial directions, and willing to consume assigned supplements (NMN or placebo) for 60 days. Exclusion criteria included currently using nicotinic acid drug or supplements, unwilling to discontinue use of conventional multivitamin/mineral or other supplements at least 2 weeks prior to the baseline visit, using statins, having used any tobacco product or recreational drugs in the past 6 months, having history of drug and alcohol abuse, having symptoms of coronavirus disease 2019 (COVID-19) as assessed by PIs, having history of unstable depression or mental illness within the last 6 months, participating or planning to begin a weight loss diet during the trial period, having hypersensitivity to the ingredients used during the trial, testing positive for urinary beta human chorionic gonadotropin or gestation period or breastfeeding for female participants, currently or within the past 30 days enrolled in another clinical trial, being unable to provide a venous blood sample, having abnormal screening laboratory test results that would indicate to be “unhealthy” in the judgment of PIs, and being unable or unwilling to provide written informed consent for participation in trial.

### Investigational product

The investigational product was food-grade NMN bulky powder with the brand name “AbinoNutra™NMN,” which was developed and manufactured by Aba Chemicals Co., Ltd. (Shanghai, China) in collaboration with Abinopharm, Inc. (Connecticut, USA) on manufacturing process development, quality control, and regulatory compliance. The two companies co-sponsored this human clinical trial. The NMN bulky powder was packed into capsules containing 150 mg NMN/capsule by Polifarma (Nanjing, China). Placebo was also produced by Polifarma as capsules of the same make, size, shape, and opaque white color filled with 150 mg/capsule rice flour. Both NMN and placebo capsules were shipped to the site of ProRelix Services, the CRO (Pune, India). The CRO did the blinding by packing the NMN or placebo capsules into opaque bottles that all labeled the same as “NMN or placebo”. Each bottle was then placed into a coded kit. A statistician at the CRO company did the randomization to evenly divide the 80 participants into 4 groups for placebo, 300 mg, 600 mg, and 900 mg NMN with 20 participants per group. The randomization code was generated and digitally locked in CRO’s database by the statistician. The statistician was the only person who was able to access and decode the randomization list.

**Fig. 1:**
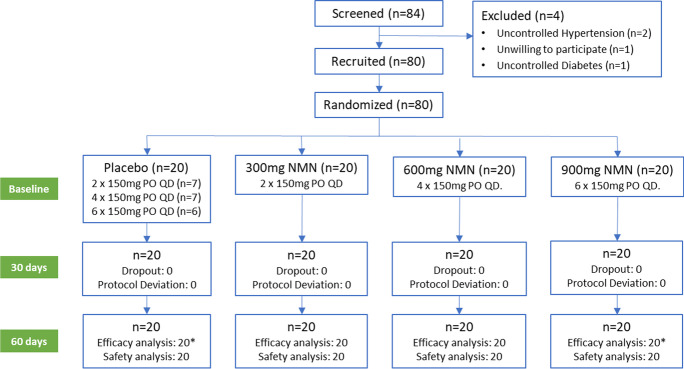
Trial flow for evaluating efficacy and safety of β-nicotinamide mononucleotide (AbinoNutra™NMN). Each recruited participant was instructed to take the assigned amount of either placebo or NMN orally once a day before breakfast with water of ambient temperature for 60 days. PO = orally, QD = once daily dosing. *Due to 1 participant each from placebo and 900 mg groups did not fast, Homeostasis Model Assessment-Insulin Resistance (HOMA-IR) was based on 19 participants for these two groups

Trial staff at the two clinical centers allocated these coded kits to participants and instructed participants to take two capsules [300 mg NMN (*n* = 20 participants) or 300 mg placebo (7 participants)], four capsules [600 mg NMN (*n* = 20 participants) or 600 mg placebo (7 participants)], or six capsules [900 mg NMN (*n* = 20 participants) or 900 mg placebo (6 participants)] a day (Fig. 1). Neither participants nor the trial staff were able to know which one of the two (NMN or placebo) was present in the bottle, thereby assuring double-blinding.

### Procedures and outcome measurements

Participants were required to visit one of the two clinic centers four times. The first visit was for screening of eligibility; second visit (day 0) for baseline assessment and randomization; third visit (day 30) for mid-point efficacy, safety, and dosing compliance assessment; and fourth visit (day 60) for end-of-trial efficacy, safety, and dosing compliance assessment.

At the 2nd visit (day 0, baseline), participants were allocated with the coded kits containing bottles of the investigational product for the entire trial and were advised to take the capsules orally with water of ambient temperature once daily before breakfast. They were instructed to maintain the record of dosing details in the participant diaries. To confirm participant’s dosing compliance with the protocol, the trial staff at the two centers checked the number of capsules dispensed and returned at 3rd visit (day 30) and 4th visit (day 60) to record the number of capsules consumed in Case Report Form (CRF), and reviewed participant dairies. Treatment compliance was considered adequate if participants had used on average at least 75% and no more than 125% of scheduled doses.

Participants could be removed from the trial at any time for any of the following reasons: participant’s withdrawal of informed consent and withdrawal by his/her own free will to not continue in the trial, participant’s non-compliance with dosing schedule (75-125% non-compliance), the development of adverse events requiring the withdrawal of the trial, and pregnancy confirmed anytime during the trial. Reasons why subjects were discontinued from the clinical trial were documented in the CRF.

#### Blood NAD concentration

Colorimetric NAD test kit from MyBioSource, Inc. (catalog# MBS841786; California, USA) was used for the measurement of NAD concentration in participants’ blood samples. The test measures the total blood concentration of NAD^+^ and NADH in serum. Therefore, the blood NAD concentration measured in our trial is the total concentration of “NAD^+^ + NADH” in the blood serum. Blood samples of each participant were taken at days 0, 30, and 60 when participants visited trial centers. The blood samples were then kept under 2-8°C and immediately transported through cold chain to a local lab of Suburban Diagnostics (Pune, India) where the samples were separated into serum within a few hours. The serum samples were then stored under −80°C before the NAD test was done according to the commercial NAD test kit’s manual.

#### Safety and tolerability

Safety evaluation of NMN supplementation was to assess if NMN would cause significant changes to clinical lab parameters from blood and urinary samples and to adverse events (AE) when compared to placebo and baseline. Clinical lab tests of blood and urinary samples were conducted at baseline and day 60. The lab clinical tests at 1st visit for screening served as baseline. Physical examination of general and systematic exam and vital signs was conducted, documented, and analyzed for safety concern at each visit. Adverse events (AE) were also monitored, recorded, and analyzed for NMN supplementation-related safety issues throughout the course of trial. Tolerability study was conducted through the comparison analysis of treatment vs. placebo on the number of dropout due to AEs.

#### Six-minute walking test

The 6-minute walking test was conducted by adapting the protocol issued by the American Thoracic Society [18] for measuring endurance and energy level. It was conducted at days 0, 30, and 60. During the 6-minute walking test, participants were asked to walk on a manual treadmill which can mimic a walking field. Distance in meters was recorded through a digital odometer of the treadmill.

#### Blood biological age

The Aging.Ai 3.0 calculator (Insilico Medicine, Inc., Hong Kong and New York) [19] based on a total of 19 clinic laboratory test parameters [albumin, glucose (fasting), urea (BUN), total cholesterol, protein total, sodium, creatine, hemoglobin, bilirubin total, triglycerides, HDL cholesterol, LDL cholesterol (by Friedewald), calcium, potassium, hematocrit, MCHC, MCV, platelets, erythrocytes (RBC)] was applied at baseline (day 0) and day 60.

#### Homeostasis Model Assessment - Insulin Resistance (HOMA-IR) test

The HOMA-IR was assessed by taking blood samples from participants when fasting at days 0 and 60. Fasting insulin and glucose levels was then measured by Suburban Diagnostics (Pune, India). HOMA-IR index was calculated by an online HOMA2 IR calculator at https://www.dtu.ox.ac.uk/homacalculator/ which is a licensed tool from University of Oxford Diabetes trial unit.

#### 36-Item Short Form Survey (SF-36 Questionnaire)

Assess participants’ overall health status or quality of life [20] was administered at days 0, 30, and 60. The total SF-36 score (37-152 points) of each participant was calculated based on participant’s responses and SF-36 scoring guideline.

#### Statistical analysis

The statistical analysis was performed using the R software (version 4.1.2). Statistical significance was set at *p* < 0.05. For baseline characteristics of participants, comparisons between treatment and placebo were analyzed by *t* test for age, weight, height, and BMI and by Chi-square *t* test for sex. For the efficacy analyses, participants without violation of trial protocol were included in the Per Protocol analyses. For blood NAD concentrations, six-minute walking test, and SF-36 scores that were measured at multiple times during the trial, mixed model for repeated measures (MMRM) was used to compare between the groups and paired *t* test was used for intragroup comparison. Paired *t* test was used for comparison of both intergroups and intragroups of blood biological age since the data were only measured at two time points (baseline and day 60) and followed normal distribution. The data from HOMA-IR did not follow normal distribution. Thus, Mann-Whitney *U* test was used for intergroup comparison while Wilcoxon signed-rank test was used for intragroup analysis. For safety and tolerability, all participants enrolled in the trial, who have received at least one dose of trial products (placebo or NMN), were included in the safety and tolerability analysis. Adverse events were presented as summaries counting both the number of separate events and the number of participants who experienced adverse events during the trial period. For clinical laboratory test data, paired *t* test was used for the statistical analysis on comparison of NMN supplementation to baseline and placebo.

## Results

### Baseline characteristics of participants

A total of 84 volunteers of were screened and 80 healthy participants with a mean age of 49.3 (SD = 6.8) years (59% female) were included in the trial (Fig. 1). Recruitment took place between May 25, 2021 and July 8, 2021. All 80 participants completed the trial without violation of the trial protocol and were included in the Per Protocol analysis except for the analysis of HOMA-IR, as two participants (one in the placebo and one in the 900 mg group) were excluded because of a non-fasting state. Participants’ baseline characteristics are shown in Table 1. All 80 participants complied with our 75-125% trial dosing protocol. The number of participants in 75-99% compliance was 7, 7, 9, and 6 respectively, while the number of participants in 100% compliance was 13, 13, 11, and 14 respectively in placebo, 300 mg, 600 mg, and 900 mg NMN groups with 20 participants in each group. There were no participants who took over 100% scheduled dose. Demographic variables were found to be evenly distributed across the placebo and three NMN groups.

**Table 1:** Participant characteristics at baseline for the placebo and three NMN-treated groups

	NMN supplementation						
	Placebo, *n* = 20^e^	300 mg, *n* = 20	600 mg, *n* = 20	900 mg, *n* = 20^e^	p 300/P^g^	p 600/P^g^	p 900/P^g^
Age (year)^a^	46.5 ± 6.7	51.2 ± 7.0	49.5 ± 6.7	49.9 ± 6.3	0.04	0.17	0.11
Female (n)^b^	12	10	14	11	0.53	0.16	0.48
Weight (kg)^a^	66.2 ± 13.5	69.2 ± 13.2	66.4 ± 10.5	66.8 ± 9.8	0.73	0.96	0.87
Height (cm)^a^	157 ± 8	159 ± 10	157 ± 10	157 ± 8	0.44	0.96	0.83
BMI (kg/m^2^)^a^	26.9 ± 4.9	27.4 ± 4.8	27.1 ± 3.9	26.9 ± 4.9	0.76	0.89	0.99
NAD (nM)^c,f^	8.11 ± 5.16	11.8 ± 11.7	7.95 ± 3.29	10.5 ± 6.8	0.20	0.91	0.22
Six-minute walking (m)^c^	325 ± 144	307 ± 108	290 ± 92	323 ± 113	0.66	0.36	0.97
Blood biological age (years)^a^	39.8 ± 7.2	42.2 ± 6.0	45.2 ± 6.5	44.3 ± 7.3	0.27	0.17	0.57
Fasting insulin (mIU/mL)^a^	18.1 ± 15.7	15.5 ± 7.2	15.1 ± 10.1	16.1 ± 9.7	0.49	0.47	0.62
Fasting glucose (mg/dL)^a^	87.1 ± 21.4	88.6 ± 8.6	94.9 ± 22.5	94.8 ± 17.3	0.77	0.26	0.21
HOMAR-IR^d^	1.41 ± 0.79	2.28 ± 1.43	1.70 ± 1.05	2.01 ± 1.26	0.25	0.94	0.43
SF-36 (score)^c^	122 ± 14	124 ± 13	118 ± 16	122 ± 17	0.95	0.40	0.95

^a^Data are “mean ± SD”. Student *t* test was used for comparison over placebo

^b^Chi-square *t* test was used for comparison over placebo

^c^Data are “mean ± SD”. Mixed model for repeated measures (MMRM) was used for comparison over placebo

^d^Data are “mean ± SD”. Mann–Whitney *U* test was used for comparison over placebo

^e^Due to 1 participant from placebo and 900 mg groups did not fast, HOMA-IR was based on 19 participants for these two groups

^f^Blood NAD concentration data are the total concentration of “NAD^+^ + NADH” in serum

^g^Statistical significance was set at *p* < 0.05

### Blood NAD concentration

**Fig. 2:**
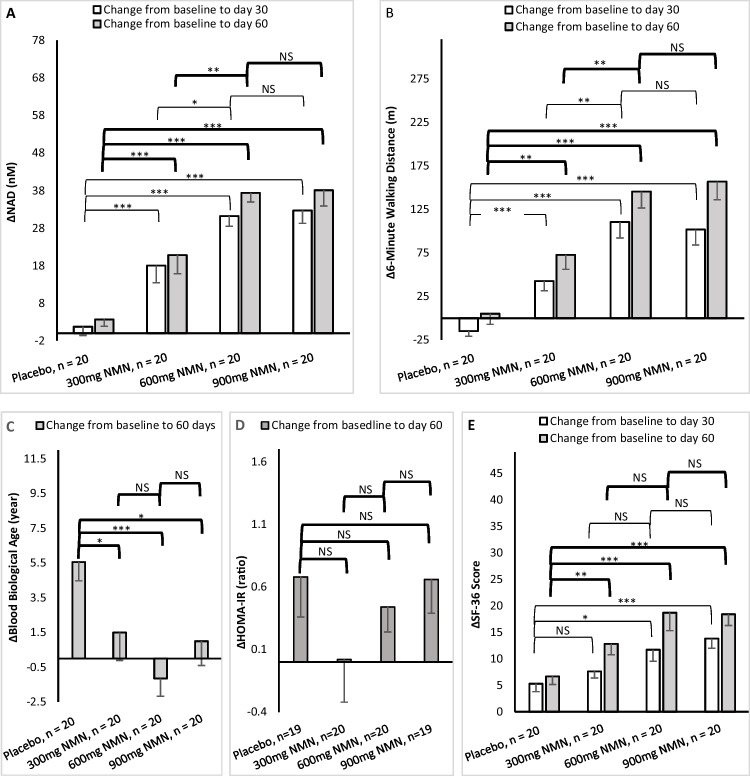
Efficacy of the placebo and three NMN-treated groups, comparisons of the three treated groups vs. placebo, 600 mg vs. 300 mg, and 900 mg vs. 600 mg on the changes of efficacy (Δmean ± SEM) from baseline to day 30 and/or day 60. **A** Comparisons on the changes of blood NAD concentrations from baseline to day 30 and day 60. **B** Comparisons on the changes of 6-minute walking distances from baseline to day 30 and day 60. **C** Comparisons on the changes of blood biological ages from baseline to day 60. **D** Comparisons on the changes of HOMA-IR ratio from baseline to day 60. **E** Comparisons on the changes of SF-36 scores from baseline to day 30 and day 60. Statistical significance is set at *p* < 0.05 (*), <0.01 (**), <0.001(***), and *p* > 0.05 = not significant (NS). Mixed model of repeated measure (MMRM) was used for blood NAD concentration, six-minute walking test, and SF-36 scores. Paired *t* test was used for blood biological age. Mann–Whitney *U* test was used for HOMA-IR. Blood NAD concentration data are the total concentration of “NAD^+^ + NADH” in serum. SEM = standard error of measurement

The NAD concentrations are given in Table 2. Blood NAD concentrations were found statistically significantly increased over baseline in all three NMN-treated groups at both days 30 and 60 (all *p* ≤ 0.001) while there were no significant changes in the placebo group over the same periods. Compared to placebo, blood NAD concentrations were statistically significantly increased for all three NMN-treated groups at both days 30 and 60 time points (all *p* < 0.001) (Fig. 2A).

**Table 2:** Efficacy of the placebo and three NMN treated groups, comparisons of treatment over baseline within the same group

	Placebo, *n* = 20^d^					300mg NMN, *n* = 20				
	Baseline	Day 30	Day 60	p1^c^	p2^c^	Baseline	Day 30	Day 60	p1^c^	p2^c^
NAD(nM)^a,e^	8.11 ± 5.16	9.83 ± 8.43	11.8 ± 9.4	0.44	0.14	11.8 ± 11.7	29.8 ± 20.1	32.6 ± 17.9	0.001	< 0.001
Six-minute walking(m)^a^	325 ± 144	310 ± 125	330 ± 117	0.73	0.90	307 ± 108	350 ± 115	380 ± 144	0.23	0.079
Blood biological age(year)^a^	39.8 ± 7.2	---	45.4 ± 8.2	---	0.029	42.2 ± 6.0	---	43.7 ± 6.7	---	0.46
HOMA-IR(ratio)^b^	1.41 ± 0.79	---	2.09 ± 1.40	---	0.048	2.28 ± 1.43	---	2.28 ± 1.42	---	0.97
SF-36(score)^a^	122 ± 14	127 ± 12	128 ± 13	0.2	0.12	124 ± 13	131 ± 12	137 ± 12	0.058	0.003
	600mg NMN, *n* = 20					900mg NMN, *n* = 20^d^				
	Baseline	Day 30	Day 60	p1^c^	p2^c^	Baseline	Day 30	Day 60	p1^c^	p2^c^
NAD(nM)^a,e^	7.95 ± 3.29	39.0 ± 12.6	45.3 ± 11.8	< 0.001	< 0.001	10.5 ± 6.8	43.1 ± 14.3	48.5 ± 19.8	< 0.001	< 0.001
Six-minute walking(m)^a^	290 ± 92	400 ± 86	435 ± 104	< 0.001	< 0.001	323 ± 113	425 ± 141	480 ± 128	0.016	< 0.001
Blood biological age(year)^a^	45.2 ± 6.5	---	44.1 ± 6.4	---	0.57	44.3 ± 7.3	---	45.3 ± 5.9	---	0.64
HOMA-IR(ratio)^b^	1.70 ± 1.05	---	2.14 ± 1.02	---	0.037	2.01 ± 1.26	---	2.67 ± 1.31	---	0.006
SF-36(score)^a^	118 ± 16	129 ± 12	136 ± 12	0.015	< 0.001	122 ± 17	136 ± 12	140 ± 11	0.005	< 0.001

^a^Data are " mean ± SD" and normally distributed. Paired t test was used for comparison over baseline

^b^Data are " mean ± SD" and are not normally distributed. Wilcoxon signed-rank test was used for comparison over baseline

^c^p1: comparison between day 30 and baseline, p2: comparison between day 60 and baseline. Statistical significance was set at *p* < 0.05

^d^Due to 1 participant each from placebo and 900mg groups did not fast, HOMA-IR was based on 19 participants for these two groups

^e^Blood NAD concentration data are the total concentration of “NAD^+^ + NADH” in serum

Compared to the 300 mg NMN group, the 600 mg NMN group had statistically significantly higher NAD concentrations at both 30-day and 60-day (*p* < 0.05 and 0.01 respectively) (Fig. 2A). No statistical difference was found between the 600 mg and 900 mg group.

### Safety and tolerability

The results of clinical lab tests are given in Table 3. Compared to the placebo group, all parameters of the three treated groups were not statistically different (all *p* > 0.05) except the results of MCHC and LDL at 900 mg NMN, and uric acid nitrogen at 600 mg NMN. There was no abnormality observed in clinical lab parameters of all participants.

**Table 3:** Clinical laboratory tests of the placebo and three NMN-treated groups, comparisons of the three NMN-treated groups to placebo at both baseline and day 60

		Placebo, *n* = 20	300 mg NMN, *n* = 20		600 mg NMN, *n* = 20		900 mg NMN, *n* = 20	
		Mean ± SD	Mean ± SD	P*	Mean ± SD	P*	Mean ± SD	P*
Hematology								
Hb, g/dL	Baseline	12.6 ± 1.8	13.5 ± 1.9	0.12	13.2 ± 2.0	0.26	13.7 ± 1.6	0.04
	Day 60	13.0 ± 1.6	13.4 ± 2.3	0.49	14.0 ± 2.2	0.10	13.9 ± 1.9	0.11
Ht-PCV, %	Baseline	38.2 ± 4.8	40.5 ± 4.3	0.12	40.2 ± 5.1	0.20	41.2 ± 4.5	0.05
	Day 60	41.3 ± 4.5	41.1 ± 6.0	0.33	43.0 ± 5.6	0.29	42.5 ± 4.8	0.41
MCHC, g/dL	Baseline	32.8 ± 1.2	33.1 ± 1.3	0.37	32.9 ± 1.2	0.83	33.2 ± 0.9	0.17
	Day 60	31.8 ± 1.2	32.3 ± 1.2	0.22	32.5 ± 1.2	0.09	32.6 ± 1.0	0.03
MCV, fL	Baseline	83.1 ± 13.0	87.6 ± 8.5	0.21	86.2 ± 6.7	0.36	88.4 ± 9.0	0.15
	Day 60	85.0 ± 12.4	91.9 ± 11.6	0.08	88.3 ± 6.8	0.31	88.1 ± 8.6	0.36
Total leukocyte count-WBC total count, /cm^2^	Baseline	7160 ± 1538	7325 ± 2132	0.78	7005 ± 1737	0.77	6780 ± 1379	0.42
	Day 60	7475 ± 1874	7835 ± 2900	0.64	7930 ± 2489	0.52	7505 ± 1721	0.96
Absolute neutrophils, /cm^2^	Baseline	4218 ± 1265	4134 ± 1559	0.85	4068 ± 1245	0.71	3806 ± 1046	0.27
	Day 60	4140 ± 1265	4275 ± 1846	0.79	4135 ± 1561	0.99	3910 ± 987	0.53
Neutrophils, %	Baseline	58.5 ± 7.7	55.5 ± 7.7	0.22	57.7 ± 8.2	0.74	55.6 ± 6.9	0.22
	Day 60	55.8 ± 7.0	53.3 ± 8.1	0.29	51.7 ± 8.1	0.09	52.3 ± 8.0	0.15
Lymphocytes, %	Baseline	34.1 ± 6.3	37.5 ± 7.4	0.13	34.5 ± 7.3	0.85	36.3 ± 6.2	0.29
	Day 60	35.4 ± 7.5	37.1 ± 7.4	0.48	39.2 ± 7.4	0.11	37.3 ± 6.1	0.38
Monocytes, %	Baseline	3.50 ± 1.02	3.80 ± 1.30	0.49	4.10 ± 1.42	0.15	3.80 ± 1.58	0.60
	Day 60	5.50 ± 1.49	5.50 ± 1.29	0.94	5.40 ± 1.53	0.93	5.50 ± 1.62	0.88
Basophils, %	Baseline	0.00 ± 0.02	0.00 ± 0.03	0.56	0.00 ± 0.02	1.00	0.00 ± 0.00	0.32
	Day 60	0.00 ± 0.02	0.00 ± 0.02	1.00	0.00 ± 0.16	0.40	0.00 ± 0.02	1.00
Eosinophils, %	Baseline	3.80 ± 3.91	3.20 ± 2.29	0.54	3.60 ± 2.89	0.89	4.40 ± 2.57	0.60
	Day 60	3.60 ± 2.78	4.20 ± 2.46	0.49	3.60 ± 2.75	0.98	4.80 ± 4.16	0.28
Platelets, /cm^2^	Baseline	289,200 ± 61,258	279,450 ± 79,343	0.67	280,450 ± 84,405	0.71	293,450 ± 77,477	0.85
	Day 60	303,900 ± 69,230	321,600 ± 86,507	0.48	311,200 ± 85,234	0.77	319,850 ± 87,594	0.53
PTT, seconds	Baseline	11.6 ± 0.6	12.3 ± 1.6	0.07	11.8 ± 0.6	0.30	11.9 ± 0.7	0.07
	Day 60	12.0 ± 0.9	12.5 ± 1.4	0.14	11.9 ± 1.0	0.91	11.9 ± 0.7	0.64
aPTT, seconds	Baseline	33.5 ± 3.1	33.0 ± 4.3	0.66	33.1 ± 5.1	0.79	32.6 ± 4.7	0.50
	Day 60	34.1 ± 6.6	32.7 ± 3.7	0.44	31.6 ± 3.8	0.16	31.1 ± 3.3	0.08
RBC, million/cm^2^	Baseline	4.70 ± 0.94	4.60 ± 0.33	0.72	4.70 ± 0.49	0.90	4.70 ± 0.60	0.99
	Day 60	4.90 ± 0.79	4.60 ± 0.69	0.16	4.90 ± 0.54	0.96	4.90 ± 0.66	0.87
Clinical blood, plasma, and serum chemistry								
TG, mg/dL	Baseline	156 ± 86	159 ± 70	0.89	157 ± 62	0.96	178 ± 83	0.41
	Day 60	161 ± 64	138 ± 72	0.29	135 ± 57	0.19	149 ± 63	0.57
LDL, mg/dL	Baseline	114 ± 30	122 ± 32	0.46	119 ± 22	0.53	132.80 ± 39.70	0.10
	Day 60	119 ± 34	116 ± 28	0.82	129 ± 26	0.29	146.00 ± 36.90	0.02
HDL, mg/dL	Baseline	42.4 ± 10.0	43.8 ± 12.3	0.69	41.8 ± 5.6	0.84	40.5 ± 8.2	0.52
	Day 60	47.9 ± 14.0	45.1 ± 15.8	0.55	45.9 ± 9.4	0.60	45.2 ± 9.1	0.48
TC, mg/dL	Baseline	171 ± 32	176 ± 32	0.60	179 ± 26	0.37	190 ± 47	0.13
	Day 60	180 ± 30	167 ± 28	0.17	185 ± 31	0.66	200 ± 41	0.10
HOMA-IR	Baseline	1.41 ± 0.79	2.28 ± 1.43	0.25	1.70 ± 1.05	0.94	2.01 ± 1.26	0.43
	Day 60	2.09 ± 1.40	2.28 ± 1.42	0.13	2.14 ± 1.02	0.89	2.67 ± 1.31	0.62
Creatine, mg/dL	Baseline	0.70 ± 0.19	0.80 ± 0.15	0.67	0.70 ± 0.15	0.72	0.80 ± 0.20	0.24
	Day 60	0.80 ± 0.20	0.80 ± 0.19	0.93	0.80 ± 0.20	0.72	0.90 ± 0.21	0.39
Urea, mg/dL	Baseline	17.6 ± 5.3	18.3 ± 5.4	0.91	18.7 ± 5.9	0.57	19.9 ± 7.1	0.25
	Day 60	18.7 ± 5.0	18.9 ± 6.1	0.91	18.5 ± 3.2	0.89	21.0 ± 7.1	0.23
AST, U/L	Baseline	23.2 ± 7.0	22.4 ± 6.2	0.71	20.9 ± 5.5	0.26	29.9 ± 21.4	0.19
	Day 60	22.4 ± 6.7	21.6 ± 7.0	0.68	22.2 ± 5.4	0.89	22.0 ± 7.2	0.84
ALT, U/L	Baseline	20.6 ± 9.2	23.7 ± 11.7	0.35	17.5 ± 6.7	0.49	32.3 ± 35.8	0.16
	Day 60	19.4 ± 7.9	19.6 ± 9.4	0.92	17.7 ± 7.0	0.49	20.2 ± 8.8	0.75
Alkaline phosphatase, U/L	Baseline	84.1 ± 22.4	83.7 ± 22.6	0.95	87.2 ± 22.2	0.67	87.2 ± 22.9	0.68
	Day 60	89.8 ± 25.1	96.6 ± 25.9	0.41	90.0 ± 26.5	0.98	94.4 ± 25.9	0.57
Bilirubin, mg/dL	Baseline	0.50 ± 0.24	0.60 ± 0.32	0.56	0.60 ± 0.38	0.42	0.50 ± 0.41	0.70
	Day 60	0.60 ± 0.23	0.60 ± 0.26	0.67	0.60 ± 0.27	0.44	0.50 ± 0.43	0.77
Sodium, mmol/L	Baseline	137 ± 4	140 ± 2	0.01	139.8 ± 2.9	0.03	139.20 ± 2.37	0.08
	Day 60	140 ± 2	140 ± 2	0.54	140.5 ± 2.2	0.31	140.3 ± 2.7	0.57
BUN, mg/dL	Baseline	8.20 ± 2.46	8.60 ± 8.25	0.67	8.7 ± 2.8	0.56	9.3 ± 3.3	0.25
	Day 60	8.80 ± 2.33	8.70 ± 2.91	0.96	8.6 ± 1.5	0.81	9.8 ± 3.3	0.26
Uric acid nitrogen, mg/dL	Baseline	5.40 ± 1.38	5.10 ± 1.21	0.61	4.5 ± 1.7	0.09	5.4 ± 1.2	0.97
	Day 60	5.50 ± 1.06	5.10 ± 1.56	0.42	4.4 ± 1.6	0.02	5.2 ± 1.3	0.46
Chloride, mmol/L	Baseline	99.6 ± 4.7	102 ± 3	0.04	102 ± 3	0.10	101 ± 3	0.19
	Day 60	102 ± 2	102 ± 3	0.36	102 ± 2	0.93	103 ± 3	0.76
Potassium, mmol/L	Baseline	4.50 ± 0.39	4.10 ± 0.25	0.002	4.30 ± 0.47	0.28	4.40 ± 0.47	0.47
	Day 60	4.70 ± 0.32	4.60 ± 0.36	0.49	4.60 ± 0.38	0.33	4.50 ± 0.42	0.10
Calcium, mg/dL	Baseline	9.20 ± 0.44	9.30 ± 0.30	0.45	9.20 ± 0.29	0.83	9.30 ± 0.42	0.45
	Day 60	9.40 ± 0.45	9.50 ± 0.40	0.40	9.40 ± 0.37	0.91	9.50 ± 0.49	0.57
GFR, mL/min	Baseline	110 ± 25	105 ± 18	0.41	99.1 ± 15.8	0.09	96.80 ± 17.63	0.05
	Day 60	100 ± 24	99.5 ± 22.7	0.90	95.50 ± 18.24	0.47	89.0 ± 16.5	0.08
Albumin, g/dL	Baseline	4.40 ± 0.29	4.40 ± 0.27	0.43	4.30 ± 0.32	0.19	4.40 ± 0.22	0.85
	Day 60	4.30 ± 0.32	4.40 ± 0.33	0.47	4.50 ± 0.35	0.17	4.40 ± 0.36	0.48
Total protein, g/dL	Baseline	7.20 ± 0.46	7.10 ± 0.41	0.30	7.20 ± 0.44	0.68	7.20 ± 0.32	0.61
	Day 60	7.50 ± 0.48	7.60 ± 0.51	0.57	7.50 ± 0.61	0.45	7.60 ± 0.54	0.76
Urinalysis								
Specific gravity	Baseline	1.00 ± 0.01	1.00 ± 0.01	0.33	1.00 ± 0.01	1.00	1.00 ± 0.00	0.03
	Day 60	1.00 ± 0.01	1.00 ± 0.00	0.14	1.00 ± 0.01	0.37	1.00 ± 0.01	0.61

*aPTT* activated partial thromboplastin time; *ALT* alanine aminotransferase; *AST* aspartate aminotransferase; *BUN* blood urea nitrogen; *GFR* glomerular filtration rate; *TC* total cholesterol; *TG* triglycerides; *Hb* hemoglobin; *Ht-PCV* hematocrit-packed cell volume; *MCHC* mean corpuscular hemoglobin concentration; *MCV* mean corpuscular volume; *LDL* low-density lipoprotein – cholesterol; *HDL* high-density lipoprotein – cholesterol; *HOMA-IR* Homeostatic Model Assessment of Insulin Resistance; *RBC* red blood cell; *PTT* prothrombin time

^*^Statistically significant difference was set at *p* < 0.05 (paired *t* test except Mann-Whitney *U* test for HOMA-IR) vs. placebo

Adverse events (AE) that occurred during the trial were summarized in Table 4. A total of nine AE cases were reported in seven participants. Six AEs were reported in five participants in placebo group and three AEs in two participants in 300 mg group. No AEs were observed in the 600 mg and 900 mg NMN-treated groups. All AEs were mild or moderate and none was related to the NMN treatment.

**Table 4:** Adverse event (AE) of the placebo and three NMN-treated groups

	Placebo, *n* = 20	300 mg NMN, *n* = 20	600 mg NMN, *n* = 20	900 mg NMN, *n* = 20
Total AEs	6 (67%)^a^	3 (33%)^b^	0	0
Serious AEs	0	0	0	0
Dropout due to AEs	0	0	0	0
AEs due to supplementation	0	0	0	0

^a^Symptoms (no. of occurrences): rashes on skin (1), tingling and numbness in all extremities (1), weakness of right upper extremity (1), irrelevant talk (1), mouth ulcer (1), and fever (1). Weakness of the right upper extremity and irrelevant talk happened on the same participant

^b^Symptoms (no. of occurrences): hyperacidity (1), skin problem (1), and mouth ulcer (1). Skin problem and mouth ulcer occurred on the same participant

No clinically meaningful abnormalities with respect to vital signs, physical findings, or any other observations were found during the trial.

### Six-minute walking test

The walking distance of the six-minute walking test is given in Table 2 and Fig. 2B. Participants in the 600 mg and 900 mg NMN-treated group walked a significantly longer distance at both 30-day and 60-day compared to baseline (all *p* < 0.05), while the 300 mg NMN group showed a trend at day 60 (*p* = 0.079). The walking performance in the placebo group did not significantly change when compared to baseline (both *p* > 0.05). Compared to placebo, the walking distances of the six-minute walking test were significantly longer for all three NMN-treated groups at both days 30 and 60 (all *p* < 0.01).

Participants in the 600 mg NMN-treated group had a statistically longer walking distance compared to the 300 mg NMN-treated group at days 30 and 60 (both *p* < 0.01). There was no statistical difference in the walking distance between the 900 mg and 600 mg NMN-treated group (both *p* > 0.05).

### Blood biological age

Results of the blood biological age are given in Table 2 and Fig. 2C. The blood biological age did not significantly change over time within the NMN-treated groups. Within the placebo group, the blood biological age increased significantly from baseline to day 60 (*p* = 0.029). When compared to placebo, the change of biological age from baseline to day 60 in the three NMN-treated groups showed statistically significant difference (all three *p* < 0.05).

### HOMA-IR index

HOMA-IR results are summarized in Table 2 and Fig. 2D. When compared to baseline, NMN treatment did not significantly change HOMA-IR in the placebo and 300 mg NMN-treated group, while HOMA-IR increased significantly in both the 600 mg and 900 mg groups. There was no statistical significance in HOMA-IR index when comparing the three treated groups to placebo or between the NMN-treated groups.

### SF-36 Questionnaire scores

SF-36 scores are summarized in Table 2 and Fig. 2E. When compared to placebo, all three NMN-treated groups at day 60 (*p* < 0.01) and 600 mg and 900 mg at day 30 (*p* < 0.05 and 0.001 respectively) gave significantly better SF-36 scores. SF-36 scores did not differ comparing between NMN-treated groups (*p* > 0.05). SF-36 scores did not change within the placebo group while the scores improved over baseline at day 60 in the 300 mg group (*p* = 0.003) and both at days 30 and 60 in the 600 mg and 900 mg NMN-treated groups (all *p* < 0.015).

## Discussion

This randomized, double-blinded, placebo-controlled trial investigated the efficacy and safety of NMN supplementation with 300 mg, 600 mg, and 900 mg daily oral doses in healthy adults of 40-65 years old. The primary objective was to evaluate blood NAD concentration. The secondary objectives were to assess the safety and tolerability of NMN supplementation, next to the evaluation of clinical efficacy by measuring six-minute walking test, blood biological age (Aging.Ai 3.0 calculator), HOMA-IR, and SF-36 scores.

In mice, oral NMN can be quickly absorbed through the intestine, efficiently transported into blood circulation, and immediately converted into NAD at various tissues, such as blood, liver, and skeletal muscle [2]. However, when we designed our trial in early 2021, it had been unclear whether and how oral NMN intervention can increase human NAD concentrations [2, 21]. The first published human clinical trials conducted in 2016 did not find that NMN supplementation increased blood NAD concentration in healthy men; instead, it only reported the increase of plasma levels of NAD metabolites [9]. A report in 2021 showed that a fixed 250 mg/day oral dosage of NMN supplementation significantly increased prediabetic and obese women’s NAD concentration in peripheral blood mononuclear cell (PBMC), but not in muscles [10]. A human clinical trial published in 2022 disclosed that a 300 mg/day oral dosage of NMN on healthy adults elevated blood NAD concentration in serum (measured as the total NAD^+^ and NADH in serum by colorimetric method) by 11.3% at day 30 and 14.3% at day 60, but the increases were statistically insignificant [11]. Another human clinical trial report disclosed a decrease of blood serum NAD concentration as measured by mass spectrometry after NMN supplementation [12]. Also published in 2022, a human clinical trial with a fixed oral dose of 250 mg/day NMN found that whole blood NAD and NMN concentrations measured by LC-MS/MS were significantly increased on healthy male participants [13]. A similar human clinical trial of NMN with a 250 mg/day fixed dosage on both healthy male and female adults was also published in 2022 and confirmed that NMN significantly increased whole blood NAD concentration, but not NMN concentration [14]. Finally, a human clinical trial published in 2022 disclosed that 1000 mg microcrystalline NMN once daily or twice daily regimens on obese participants for 14 days statistically significantly and dose-dependently increased both NMN and NAD concentrations in whole blood as measured by LC-MS/MS [15]. As seen in the above reported seven human clinical trials of NMN supplementation on the effect of blood NAD concentrations, the results are mixed but overall confirmed that orally administered NMN can be absorbed by the human digestive system into systemic circulation and quickly converted into NAD in human blood. The particle size and crystalline forms of NMN investigational product, the ways of blood sample collection, and the analytical methods of blood samples affect the results of final blood NAD concentrations [14]. Our investigational product was AbinoNutra™NMN, a fine crystalline powder, which increased blood NAD concentrations significantly in a dose-dependent manner. Blood NAD concentration was measured as the total “NAD^+^ + NADH” concentration in serum by validated commercial colorimetric method.

This trial showed that NMN supplementation is safe and well tolerated at up to 900 mg oral daily doses. There were no NMN treatment-related adverse events and dropouts. Lab parameters and physical examination did not show significant abnormal changes during the 60-day NMN treatments of all three doses. Our safety and tolerability observations are consistent with results of other published human clinical trials [9–17].

It was reported that NMN supplementation in aged mice increased NAD concentrations and improved physical activity [1], and increased treadmill running distance significantly by activating SIRT1 in skeletal muscle vascular endothelial cells [6]. When compared to placebo, our six-minute walking test results showed an increase of distances covered during the 6 min for all three NMN doses and the two time points except the 300 mg group at day 30, which is consistent with other published human clinical trials that NMN supplementation can increase skeletal muscle metabolization function [10], muscle strength [13], muscle utilization of oxygen [16], and lower limb function [17]. However, one trial reported that there was no statistically significant difference in its six-minute walking test when compared to placebo [11]. Finally, the results of our six-minute walking test are right within the published range of 294-691 m of healthy Indian adults considering all our trial participants are Indian ethnicity [22].

Biological age has been gaining attention including epigenetic clock and blood biological age [23, 24]. Biological age clock is used to predict a person’s lifespan and healthspan [23]. The Aging.Ai 3.0 model developed by Insilico Medicine was used for our blood biological age study [19]. This biological age clock, also called hematological clock or blood biological age, uses 19 blood test parameters for calculating the blood biological age. Our trial found that NMN supplementation helped maintain the blood biological age while it increased significantly in placebo during the 60-day trial.

One previous human clinical trial reported that NMN supplementation can increase the skeletal muscle insulin sensitivity of prediabetic and obese female adults [10]. HOMA-IR has been widely used for the estimation of insulin sensitivity [25]. Our trial found that NMN supplementation has no effect on HOMA-IR of healthy adults. Similar results were also reported from two other human clinical trials [11, 13].

Previous human clinical trials reported that NMN supplementation had no effect on sleep quality [9, 17], no impact on age-related phenotypes [sensory as measured by hearing improvement, vascular as evaluated by blood pressure and flow-mediated dilation, and cognition as assessed by using mini-mental state examination-Japanese (MMSE-J) and Japanese version of the Montreal Cognitive Assessment (MOCA-J)] [13], and mixed results on fatigue [17]. One previously published clinical trial in healthy adults reported that NMN supplementation did not generate significant improvement on SF-36 scores as a measure for overall health [11]. The results from this trial on SF-36 assessment indicated that the NMN supplementation improved middle-aged adults’ overall health status at day 60, and higher doses and longer NMN treatment gave better SF-36 scores.

This trial was completed without dropout and by strictly following our trial protocol and GCP guidelines. We achieved positive results on many of our trial end points on efficacy and safety of NMN supplementation. However, an even higher number of participants and longer trial duration might have given even more insightful results of trial end points. Furthermore, additional biological age clocks and longer trial duration are needed to confirm our results. It will also be very interesting to conduct a larger study to assess the impact of gender on many of our trial end points since males and females could respond differently to NMN supplementation. Finally, our trial measured the total “NAD^+^ + NADH” concentration in serum. Future studies directed to measure NAD concentration in whole blood or other tissues will help gain more comprehensive values about the impact of NMN supplementation.

In conclusion, blood NAD concentration was significantly and dose-dependently increased during the NMN treatment. Oral administration of NMN up to 900 mg/day for 60 days was safe and well tolerated. NMN supplementation had a positive impact on the physical endurance and general health conditions of healthy adults as demonstrated in the significant improvement of six-minute walking test, blood biological age, and SF-36 scores. The 900 mg/day oral dose did not give significantly better efficacy than 600 mg/day dose.

## Supplementary Information

Below is the link to the electronic supplementary material.

Supplementary file1 (PDF 162 KB)

## Data Availability

All data will be available on reasonable request to the corresponding author. A proposal will be needed for assessment of request.
