# Supplementary material for: The efficacy and safety of β-nicotinamide mononucleotide (NMN) supplementation in healthy middle-aged adults: a randomized, multicenter, double-blind, placebo-controlled, parallel-group, dose-dependent clinical trial
Source: GeroScience. 2022 Dec 8;45(1):29–43. doi: 10.1007/s11357-022-00705-1 (PMC9735188; doi:10.1007/s11357-022-00705-1)
Supplement: Supplementary file 1 — Supplementary file1 (PDF 162 KB) [file 11357_2022_705_MOESM1_ESM.pdf]

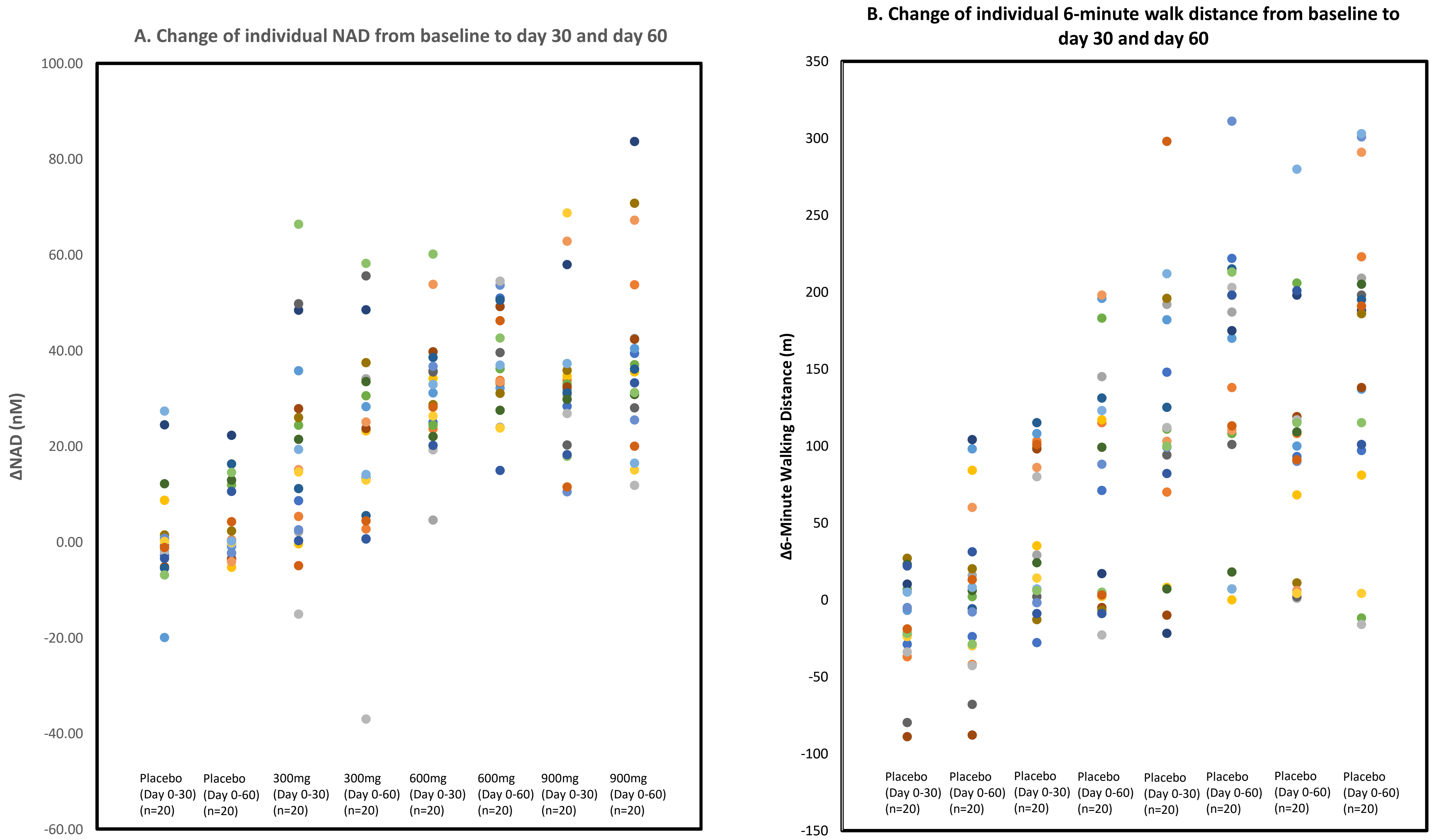

A. Change of individual NAD from baseline to day 30 and day 60
ΔNAD (nM)
100.00
80.00
60.00
40.00
20.00
0.00
-20.00
-40.00
-60.00
Placebo (Day 0-30) (n=20)
Placebo (Day 0-60) (n=20)
300mg (Day 0-30) (n=20)
300mg (Day 0-60) (n=20)
600mg (Day 0-30) (n=20)
600mg (Day 0-60) (n=20)
900mg (Day 0-30) (n=20)
900mg (Day 0-60) (n=20)
B. Change of individual 6-minute walk distance from baseline to day 30 and day 60
Δ6-Minute Walking Distance (m)
350
300
250
200
150
100
50
0
-50
-100
-150
Placebo (Day 0-30) (n=20)
Placebo (Day 0-60) (n=20)
Placebo (Day 0-30) (n=20)
Placebo (Day 0-60) (n=20)
Placebo (Day 0-30) (n=20)
Placebo (Day 0-60) (n=20)
Placebo (Day 0-60) (n=20)
Placebo (Day 0-60) (n=20)

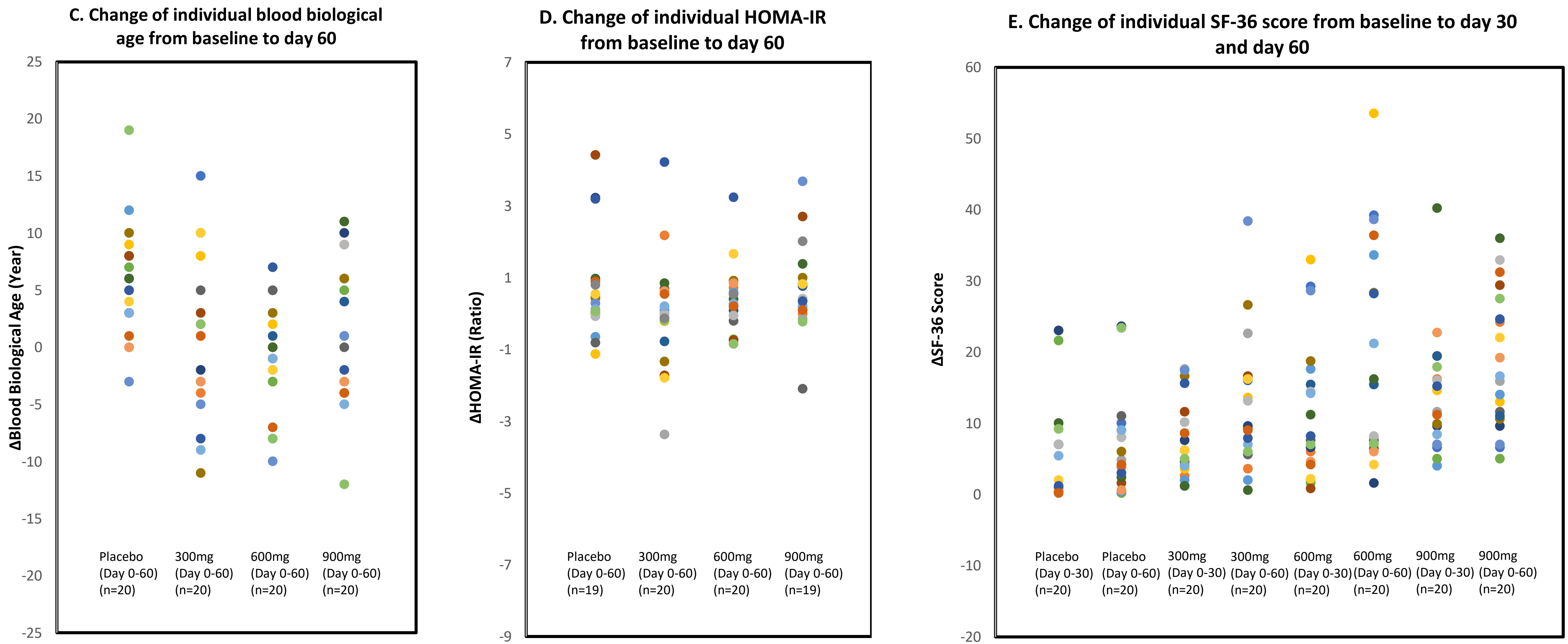

Supplemental Fig. 1 Change of individual efficacy from baseline to day 30 and/or day 60 for the placebo and three NMN-treated groups. **A** Change of individual blood NAD concentration from baseline to day 30 and day 60. **B** Change of individual 6-minute walking distance from baseline to day 30 and day 60. **C** Change of individual blood biological age from baseline to day 60. **D** Change of individual HOMA-IR from baseline to day 60. **E** Change of individual SF-36 score from baseline to day 30 and day 60.
